# Sustainable valorization of fish viscera into omega-3 rich lipids and their functional validation

**DOI:** 10.1186/s13568-026-02029-1

**Published:** 2026-02-20

**Authors:** K. Bhargav Reddy, Anjani Devi Chintagunta, Ravi Kumar Gutti, N. S. Sampath Kumar

**Affiliations:** 1 Department of Biotechnology, School of Biotechnology and Pharmaceutical Sciences, Vignan’s Foundation for Science, Technology and Research, Vadlamudi, Andhra Pradesh 522213 India; 2 Department of Biochemistry, School of Life Sciences, University of Hyderabad, Hyderabad, Telangana 500046 India

**Keywords:** Fish viscera valorization, Polyunsaturated fatty acids, Green solvent extraction, Urea complexation, Bioactivity assays, Circular bioeconomy

## Abstract

Fish-processing generates substantial viscera waste that is often discarded or sold at negligible prices, representing both an environmental burden and a missed economic opportunity. This study demonstrates the sustainable valorization of *Catla catla* viscera through green solvent extraction and urea complexation to obtain polyunsaturated fatty acid (PUFA)-rich fractions. Among extraction methods, the autoclave-assisted approach gave the highest lipid yield (16 ± 0.7%), while the classical Bligh & Dyer method preserved the most balanced PUFA profile (20.16%, with EPA 9.47% and DHA 6.82%). Green-solvent modifications (2-MeTHF and ethanol) maintained yields (~ 9.9%) while enhancing ω-3 PUFA recovery to 19.80%. Subsequent urea complexation increased total PUFA from 28.44% to 56.03% and ω-3 fractions to 49.86% at a 6:1 molar ratio. The PUFA-enriched fraction demonstrated measurable in vitro bioactivities, including antioxidant capacity (IC₅₀ < 0.2 mg mL⁻¹), enhanced wound closure of 78.79 ± 3%, and significant immunomodulation (TNF-α reduced to 112.68 pg/mL; TGF-β1 increased to 571.52 pg/mL) at 50% concentration. A preliminary economic estimation suggests that valorization of viscera into PUFA-rich lipids may substantially enhance value compared with raw disposal, although detailed techno-economic analysis and pilot-scale validation are required to confirm feasibility. This dual demonstration of bioactivity and valorization potential highlights viscera-derived lipids as promising nutraceutical and biomedical resources.

**Supplementary Information:**

The online version contains supplementary material available at 10.1186/s13568-026-02029-1.

## Introduction

Aquaculture in India is primarily promoted to meet the growing demand for affordable protein and essential nutrients, with freshwater fish playing a central role in nutritional security (Barik 2017). Within inland aquaculture, Indian major carps (IMCs) dominate, and *Catla catla (Catla*) is one of the most widely cultured freshwater species, representing about 4–5% of global freshwater aquaculture output (FAO, 2022). However, large-scale farming and processing of these fish inevitably generate substantial by-products, with 30–70% of total biomass potentially discarded as waste, depending on the species and processing method. Viscera alone account for 10–18% of fish weight depending on species and season (Swapna et al. 2010). Despite being rich in proteins, peptides, lipids, and digestive enzymes, viscera are often discarded without utilization, resulting in economic losses and environmental concerns. Valorization of such side-streams aligns with circular bioeconomy principles by enabling the recovery of polyunsaturated fatty acids (PUFA) enriched oils and other bioactives, while reducing waste burdens and supporting sustainable aquaculture practices (Alfio et al. 2021).

Among fish-processing by-products, PUFAs are of particular interest due to their health-promoting properties (Yoshida et al. 2003). Long-chain ω-3 PUFAs such as eicosapentaenoic acid (EPA) and docosahexaenoic acid (DHA) are known for their antioxidant, antimicrobial, wound healing, and anti-inflammatory activities (Paul et al. 2018; McDaniel 2021). At present, marine fishes remain the primary commercial source of these fatty acids, but dependence on marine resources raises concerns related to sustainability and seasonal variations. In contrast, cultured freshwater fishes, especially *Catla catla* may provide a more consistent and predictable supply of PUFA-rich lipids (Li et al. 2011).

Given the nutritional and therapeutic value of PUFA, efficient extraction and enrichment strategies are necessary to recover them from fish-processing wastes and validate their biological potential. Lipid recovery from fish-processing by-products has been attempted through different approaches, such as solvent-based, physical, thermal, and advanced methods including supercritical CO₂ extraction and enzyme-assisted processes (Adeoti and Hawboldt 2014). Among conventional methods, the classical Bligh and Dyer protocol (Bligh and Dyer 1959) remains a benchmark for lipid extraction, although its reliance on chloroform and methanol raises environmental and safety concerns.

To address this limitation, green solvents such as ethanol and 2-methyltetrahydrofuran (2-MeTHF) have recently gained attention as sustainable alternatives (Prasad et al. 2022). In parallel, physical assistance methods such as ultrasound-assisted extraction (UAE) and autoclave-assisted extraction (AAE) have been explored to improve lipid recovery efficiency, though these may influence fatty acid composition and stability (Lakmini et al. 2022; Ciftci and Keskin Cavdar 2025). For selective enrichment of PUFA, several methods have been reported, including low-temperature crystallization, molecular distillation, supercritical fluid extraction, and enzymatic fractionation (Yi et al. 2023). Among these, urea complexation is widely recognized as a simple, cost-effective, and scalable technique for separating PUFA from saturated and monounsaturated fatty acids (Eskandari et al. 2024). Several studies have shown that enriched PUFA fractions exhibit measurable biological activities, which are commonly evaluated through antioxidant, antimicrobial, cytotoxicity, wound healing, and anti-inflammatory assays. However, systematic investigations linking PUFA enrichment and functional validation from freshwater aquaculture species in India, such as *Catla catla* studies are limited.

Addressing this gap through simple and scalable valorization strategies could enable recovery of high-value lipids while supporting waste reduction and local bioresource utilization. Accordingly, the primary objective of this study was to develop a sustainable and scalable strategy for recovering ω-3 PUFA-rich lipids from *Catla catla* viscera using green solvent extraction and urea complexation, and to functionally validate the enriched fraction using representative in vitro bioassays. Comparative extraction methods and economic assessment were included to contextualize feasibility rather than to optimize each parameter independently.

## Materials and methods

### Materials

All chemicals and reagents were of analytical reagent (AR) or cell culture grade. Methanol, chloroform, ethanol, n-hexane, 2-methyltetrahydrofuran (2-MeTHF), boron trifluoride (BF₃) in methanol, sodium hydroxide, potassium hydroxide, hydrochloric acid, phosphate-buffered saline (PBS), phosphate-buffered saline with Tween-20 (PBST), and butylated hydroxytoluene (BHT) were procured from Sisco Research Laboratories Pvt. Ltd. (Mumbai, India). The standard mixture of fatty acid methyl esters (C8–C24) was obtained from Sigma-Aldrich (USA). Reference compounds included ascorbic acid, ampicillin, ciprofloxacin, doxorubicin, cordycepin, and cipladine (all analytical grade). Dulbecco’s Modified Eagle Medium (DMEM), keratinocyte growth medium, fetal bovine serum (FBS), and antibiotic–antimycotic solution were purchased from Gibco (Thermo Fisher Scientific, USA). Commercial ELISA kits for TNF-α and TGF-β1 quantification were obtained from Elabscience (USA). All solvents and reagents were of high-purity grade and stored according to manufacturer instructions until use.

### Sample collection and preparation

A total of 21 cultured *Catla catla* specimens (common name: *catla*/ bocha) were sourced from a freshwater aquaculture pond via a local vendor in Tenali, Andhra Pradesh, India (16.2379° N, 80.6444° E). The average body weight of the specimens was 6.55 ± 1.36 kg, with a mean body length of 59.8 ± 1.22 cm and width of 22 ± 3.1 cm. The fish were transported immediately after harvest in insulated iceboxes and processed within 2 h to preserve tissue integrity. Upon arrival at the laboratory, viscera were separated and weighed. Viscera from all 21 fish were pooled and thoroughly homogenized to ensure compositional uniformity, and the homogenate was then subdivided into aliquots for extraction experiments. Each extraction method was applied to aliquots drawn from the same homogenized pool to minimize biological variation. All extractions were performed in triplicate as technical replicates. The homogenized samples were divided into 500 g aliquots, placed in sterile collection bags, and stored at − 20 °C until further use.

### Lipid extraction

#### Bligh and dyer extraction

Total lipids were extracted using the classic Bligh and Dyer protocol (Bligh and Dyer 1959). Homogenized viscera (50 g) were treated with chloroform and methanol (2:2, v/v) to form a monophasic system for solubilizing polar and non-polar lipids. The mixture was vigorously mixed for 5 min. Distilled water was then added to achieve a final solvent ratio of 2:2:1.8 (v/v/v; chloroform: methanol: water), forming a biphasic system. The lower chloroform-rich phase containing the lipids was collected, dried, and stored.

#### Ultrasound-assisted extraction (UAE)

Fifty grams of homogenized viscera were placed in a solvent system (chloroform: methanol; (2:2 v/v) and exposed to ultrasound at 40 °C for 30 min. The power was maintained at 40% of maximum output, with a frequency of 30 kHz. The extract was centrifuged to remove debris, and the solvent was evaporated. Lipids were collected and stored for further use (Keskin Çavdar et al. 2023).

#### Autoclave-assisted extraction (AAE)

Following a modified method of Lakmini et al. (2022), 50 g of homogenized viscera were placed in a glass vessel, sealed with aluminium foil, and autoclaved at 121 °C for 30 min. The aqueous phase was filtered through muslin cloth and centrifuged at 4400 × g for 15 min at 25 °C. The lipid-rich upper layer was collected and stored at –20 °C.

#### Green solvent-based modified bligh and dyer method

A green modification of the Bligh and Dyer extraction was carried out using three solvent systems. In Set 1, chloroform was retained while methanol was replaced by ethanol. In Set 2, chloroform was replaced with 2-methyltetrahydrofuran (2-MeTHF) while methanol was retained. In Set 3, both chloroform and methanol were replaced with 2-MeTHF and ethanol, respectively. For all sets, solvents were added in a 2:2 ratios (v/v) to the homogenized viscera and vortexed for 5 min. Distilled water was then added to achieve a final solvent ratio of 2:2:1.8 (v/v/v). The mixtures were centrifuged at 3000 × g for 10 min at 4 °C to promote phase separation. The organic phase was recovered (lower layer in Set 1; upper layer in Sets 2 and 3), evaporated, and stored at − 20 °C until analysis. Ethanol and 2-MeTHF were chosen for their low toxicity, biodegradability, and recyclability, consistent with green chemistry principles (Prasad et al. 2022).

#### Fatty acid characterization by GC-FID

Fatty acid composition was determined using gas chromatography–flame ionization detection (GC-FID) after derivatization to FAMEs (Morrison and Smith 1964; Abdolshahi et al. 2015). Briefly, 200 µL of lipid extract was reconstituted in 2 mL of 1 N methanolic NaOH, vortexed, and heated at 60 °C until clear. Three millilitres of 14% boron trifluoride (BF₃) in methanol were added, vortexed, and heated at 70 °C for 15 min. After cooling, 1 mL water and 1 mL n-hexane were added for extraction. The upper organic layer was collected, dried over anhydrous sodium sulfate, and analyzed on an Agilent 7890B GC system equipped with a DB-WAX capillary column (30 m × 0.25 mm, ID,0.25 µm film thickness). One microlitre of sample was injected in split mode (split ratio 10:1). Oven program: initial 60 °C (5 min hold), ramp 4 °C/min to 240 °C, hold 15 min. Helium was used as the carrier gas at 2.0 mL/min. Injector and detector temperatures were maintained at 250 °C and 300 °C, respectively. Fatty acids were identified by comparing retention times with authentic FAME standards (C8–C24, Sigma-Aldrich). Data acquisition and peak integration were performed using Agilent ChemStation software. An integration threshold of 0.1% of the total peak area was applied to exclude noise and trace impurities (Chiu and Kuo 2020; Yang et al. 2024).Fatty acid composition is expressed as g per 100 g of total identified fatty acids.

#### Urea complexation

Crude lipid (5 g) was saponified in 20 mL of 95% aqueous ethanol containing 1.5 g KOH at 65 °C for 2 h. After separation of the non-saponifiable fraction, the saponified solution was acidified to pH 1–3 using 3 N HCl. FFAs were extracted with 30 mL n-hexane, evaporated under reduced pressure, and stored (Ishak et al. 2019)**.** For enrichment, urea:FFA molar ratios of 2:1, 4:1, 6:1 and 8:1 was tested. Urea was dissolved in 40 mL of ethanol at 60 °C. FFA’s (5 g) with 0.01 g BHT were added, stirred until fully dissolved, then cooled gradually to room temperature and refrigerated at 4 °C for 12 h to induce crystallization. Crystals were separated by vacuum filtration, and the PUFA-rich filtrate was acidified (pH 4–5, 6 N HCl) before extraction with 30 mL n-hexane. The organic phase was collected, dried and stored for GC-FID analysis (Eskandari et al. 2024).

####  Biological activity assessment

Lipid samples were prepared as homogeneous emulsions by vigorous vortexing immediately prior to dilution in the respective assay media. Visual inspection confirmed the absence of phase separation during assay incubation. Concentrations were expressed in percentage or mass-based units depending on the assay.

#### Antimicrobial activity (MIC)

The antibacterial activity of crude lipid and PUFA-enriched fractions were assessed by broth microdilution in 96-well microplates against representative Gram-positive and Gram-negative bacteria (*Staphylococcus aureus* (MTCC 9760), *Escherichia coli* (MTCC 443), *Pseudomonas aeruginosa* (MTCC 3541) and *Vibrio alginolyticus* (MTCC 13127)) were employed for assessment. Serial two-fold dilutions of the samples (range: 256–0.5 mg mL⁻^1^) were prepared in Mueller–Hinton broth. Inocula were adjusted to approximately 1 × 10⁶ CFU/mL (0.5 McFarland standard). Wells containing only medium served as negative controls, and wells with inoculum but no sample were used as positive growth controls. Ampicillin (10 µg/mL) was used as a positive control for Gram-positive bacteria, while ciprofloxacin (10 µg/mL) served as a reference standard for Gram-negative bacteria. Plates were incubated at 37 °C for 24 h. Bacterial growth inhibition was quantified spectrophotometrically, and inhibitory concentration values corresponding to 50% growth reduction (MIC₅₀) were determined. All assays were performed in triplicate (European Committee for Antimicrobial Susceptibility Testing (EUCAST) of the European Society of Clinical Microbiology and Infectious Diseases (ESCMID) 2003).

#### Antioxidant activity (DPPH assay)

The antioxidant activity of crude lipid and PUFA-enriched fractions was determined using the 2,2-diphenyl-1-picrylhydrazyl (DPPH) assay (Blois 1958). A 0.1 mM DPPH solution was prepared in methanol and kept in the dark until use. Serial dilutions of the PUFA-enriched fractions were prepared in methanol. For each reaction, 1 mL of the sample solution was mixed with 2 mL of DPPH solution, vortexed, and incubated at room temperature in the dark for 30 min. The decrease in absorbance was measured at 517 nm using a UV–Visible spectrophotometer. A methanol–DPPH mixture without sample served as the control, while ascorbic acid was used as the positive reference standard. The scavenging activity was calculated as:

$$ {\text{Scavenging activity }}\left( {{\% }} \right){ } = { }\left[ {\left( {{\mathrm{A}}_{0} { } - {\text{ A}}_{s} } \right){ }/{\text{ A}}_{0} } \right]{ } \times { }100 $$

where A_0_= absorbance of control and A_s_ = absorbance of sample.

#### Cytotoxicity assay (MTT)

The cytotoxicity of crude lipid and PUFA-enriched fractions was assessed using the 3-(4,5-dimethylthiazol-2-yl)-2,5-diphenyltetrazolium bromide (MTT) assay (Gerlier and Thomasset 1986) in two cell lines: normal human epidermal keratinocytes (NHEK; Innoprot, Spain) and murine macrophages (RAW 264.7; National Centre for Cell Sciences, Pune, India). NHEK cells were maintained in keratinocyte-specific medium, and RAW 264.7 cells were cultured in Dulbecco’s Modified Eagle Medium (DMEM) supplemented with 10% fetal bovine serum (FBS) and 1% antibiotic–antimycotic solution at 37 °C in a humidified atmosphere containing 5% CO₂. Cells were seeded in 96-well plates at a density of 1.5 × 10^4^ cells/well and allowed to adhere for 24 h. Test samples were prepared in culture medium at concentrations of 3.125%, 6.25%, 12.5%, 25%, and 50%. After 24 h of treatment, the medium was replaced with fresh medium containing MTT reagent (0.5 mg mL⁻^1^), and plates were incubated in the dark for 3 h. The medium was then discarded, and the resulting formazan crystals were dissolved in dimethyl sulfoxide (DMSO). Absorbance was recorded at 570 nm using a microplate reader. Controls included: (i) medium-only (blank), (ii) untreated cells (negative control), (iii) doxorubicin (1 µg/mL) as positive control for NHEK cells, and (iv) lipopolysaccharide (LPS, 1 µg/mL) as positive control for RAW 264.7 cells. Cell viability at each lipid concentration was calculated relative to untreated control cells cultured in medium alone, enabling assessment of direct cytotoxic effects of the lipid fractions. Cell viability was expressed using the formula:

$$Cell viability \left(\%\right)= \frac{Absorbance of treated cells}{Absorbance of untreated cells } \times 100$$

#### In vitro wound scratch assay

The wound healing activity of crude lipid and PUFA-enriched fractions were evaluated using normal human epidermal keratinocytes (NHEK-293) cells at passage 24. Cells were seeded in 12-well plates at a density of 0.25 × 10⁶ cells/well and cultured to 80–100% confluence over 24 h. A sterile 200 µL pipette tip was used to create a uniform scratch across the monolayer, followed by gentle washing with fresh medium to remove detached cells (Jonkman et al. 2014).Test samples were added at 50% concentration in complete medium. Cipladine (50 µg/mL) was included as a positive control, and untreated wells served as negative controls. Plates were incubated for 48 h under standard culture conditions (37 °C, 5% CO₂). Microscopic images were captured at 0 h and 24 h using an inverted microscope under identical settings. Wound areas were quantified using ImageJ software, with three fields analyzed per well. Each treatment was performed in triplicate (n = 3). The percentage of wound closure was calculated as:

$$W\text{ound closure }(\mathrm{\%})= \frac{\text{Initial wound area}-\text{ Final wound area}}{\text{Initial wound area}}\times 100$$

#### Anti-inflammatory assay

The anti-inflammatory assay of PUFA-enriched fractions was conducted in LPS-stimulated RAW 264.7 macrophages, a well-established model for cytokine induction (Zhang et al. 2014). Cells were seeded in 6-well plates at a density of 0.5 × 10⁶ cells/well in 2 mL of complete medium and incubated overnight at 37 °C. Inflammation was induced by lipopolysaccharide (LPS, 1 µg/mL) for 2 h, after which cells were treated with the test samples at concentrations of 3.125–50% and incubated for 24 h. Controls included: (i) medium-only (blank), (ii) untreated cells (negative control), (iii) LPS-induced cells (positive inflammatory control), and (iv) LPS-induced cells treated with cordycepin (30 µg/mL) as a pharmacological standard. After incubation, cells were washed with PBS and lysed with 1% PBST buffer, followed by storage at –80 °C for 2 h. Lysates were harvested by scraping and centrifuged at 1900 × g for 10 min at 4 °C, and supernatants were collected for cytokine analysis. Levels of Tumor Necrosis Factor-alpha (TNF-α, pro-inflammatory) and Transforming Growth Factor-beta 1 (TGF-β1, anti-inflammatory) were quantified using commercial ELISA kits (Elabscience, USA) according to the manufacturer’s protocol. Briefly, samples and standards were added to antibody-coated wells, followed by incubation, washing, and sequential exposure to biotinylated detection antibody, HRP-conjugated streptavidin, and TMB substrate. Reactions were stopped with acid, and absorbance was measured at 450 nm using a microplate spectrophotometer. Cytokine concentrations were determined from standard curves and expressed as mean ± standard deviation (SD) from triplicate experiments.

#### Statistical analysis

All experiments were performed in triplicates (n = 3). Data were expressed as mean ± SD. Statistical analyses were conducted using SPSS Statistics (V 25.0). Differences among treatment groups were analyzed by one-way analysis of variance (ANOVA), appropriate for comparing more than two groups, followed by Tukey’s post hoc test to identify pairwise differences. A probability value of *p* < 0.05 was considered statistically significant.

## Results

### Extraction yield of lipids

The efficiency of lipid extraction varied considerably among the three methods employed. The Bligh and Dyer method yielded 11.14 ± 0.5% crude lipids, a value consistent with or slightly higher than previous reports. The ultrasound-assisted extraction (UAE) method produced 12.9 ± 0.6%, exceeding the Bligh and Dyer recovery. The UAE method showed a higher lipid recovery compared to the solvent-based extraction under the conditions employed (Carreira-Casais et al. 2021). The autoclave-assisted method yielded the highest recovery at 16 ± 0.7%, representing the maximum lipid yield among the three extraction approaches evaluated.

### Characterization of fatty acids

**Fig. 1:**
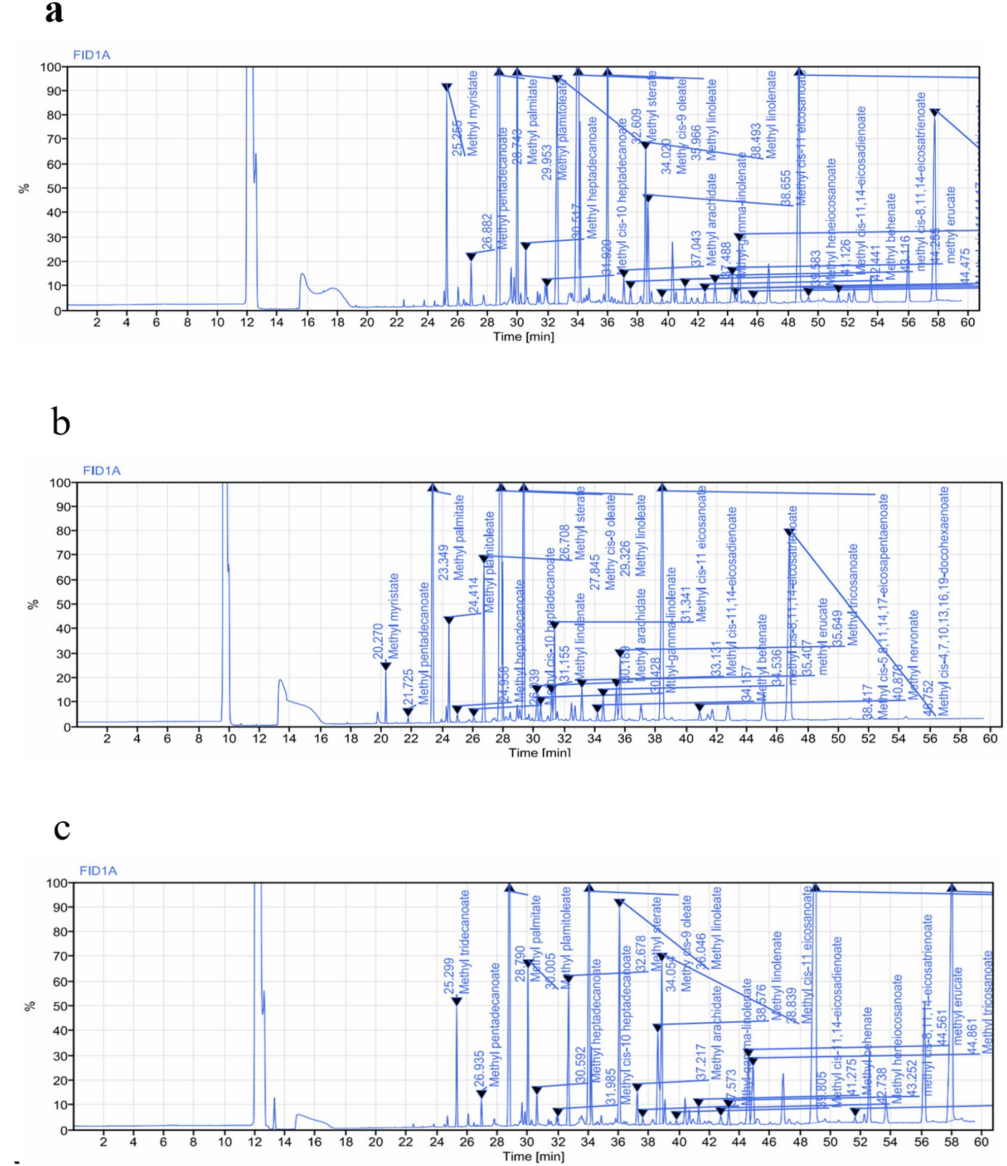
GC–FID chromatograms illustrating fatty acid profiles at key stages of the valorization workflow: (**a**) conventional Bligh & Dyer extraction, (**b**) green-solvent-modified extraction, and (**c**) PUFA-enriched fraction obtained by urea complexation (6:1 urea:FFA molar ratio)

The fatty acid composition of lipids extracted from *Catla catla* viscera was analysed by GC-FID after conversion to FAMEs. This enabled quantification of saturated (SFA), monounsaturated (MUFA), and polyunsaturated fatty acids (PUFA). Table 1 summarizes fatty acid composition of lipid fractions extracted via Bligh & Dyer, ultrasound-assisted (UAE), and autoclave-assisted methods. Among these methods, the conventional Bligh & Dyer (B&D) extraction yielded a well-balanced fatty acid profile, with a total PUFA content of 28.44 g/100 g, of which ω-3 fatty acids accounted for 20.16 g/100 g. Importantly, B&D extraction enabled efficient recovery of long-chain ω-3 PUFA, including EPA (9.47 g/100 g) and DHA (6.81 g/100 g), highlighting its effectiveness in extracting nutritionally relevant polyunsaturated fatty acids from visceral tissues (Fig. 1a). UAE and autoclave extractions showed contrasting fatty acid patterns. The UAE sample exhibited the highest MUFA content (41.64 g/100 g), dominated by oleic acid (C18:1) at 36.23 g/100 g.

**Table 1:** Fatty acid composition of lipid fractions extracted using Bligh & Dyer, ultrasound-assisted, and autoclave-assisted methods

Category	Name of the fatty acid		Bligh and Dyer(g/100 g)	UAE(g/100 g)	Autoclave (g/100 g)
Saturated fatty acids (SFA)	Myristic Acid	C14:0	3.28	2.52	7.05
	Palmitic Acid	C16:0	22.69	21.04	28.94
	Stearic Acid	C18:0	6.05	5.18	8.87
	Arachidic Acid	C20:0	0.55	0.21	< 0.1
	Lignoceric Acid	C24:0	< 0.1	< 0.1	< 0.1
Monounsaturated fatty acids (MUFA)	Palmitoleic Acid	C16:1	2.23	4.06	10.30
	Oleic Acid	C18:1	0.22	36.23	14.72
	Eicosenoic Acid	C20:1	2.23	1.34	1.47
Polyunsaturated fatty acids (PUFA)	Linolenic Acid	C18:3n3	3.77	0.49	6.00
	EPA	C20:5n3	9.47	< 0.1	2.19
	DHA	C22:6n3	6.81	5.56	4.78
	Linoleic Acid	C18:2n6	6.94	18.80	9.18
	GLA	C18:3n6	0.31	0.54	< 0.1
	ARA	C20:4n6	0.12	< 0.1	< 0.1

The total PUFA level was 27.44 g/100 g, of which ω-3 PUFA accounted for 6.05 g/100 g. EPA was not detected, while DHA was found to be 5.56% at RT 47.03 min (Fig S1). In contrast, autoclaving yielded the highest SFA (51.26 g/100 g, dominated by palmitic acid, 28.94 g/100 g). EPA (2.19%) and DHA (4.78%) were detected, but the total PUFA content was lower (12.97 g/100 g). Collectively, ultrasound-assisted and autoclave-assisted extractions are presented as comparative benchmarks to contextualize lipid recovery and fatty acid distribution, while the Bligh & Dyer–based workflow was selected for subsequent green-solvent modification and PUFA enrichment.

### Green-solvent modified bligh & dyer extraction

Although the conventional Bligh & Dyer method yielded a balanced fatty acid profile with substantial ω-3 PUFA enrichment, its dependence on chloroform and methanol poses significant environmental and occupational safety hazards. To address this, the protocol was modified by replacing chloroform with 2-methyltetrahydrofuran (2-MeTHF), a biomass-derived ether considered a green alternative solvent, and methanol with ethanol, a food-grade, low-toxicity alcohol. Both 2-MeTHF and ethanol are biodegradable, renewable, and widely accepted in food and pharmaceutical applications, aligning with the principles of green chemistry (Prasad et al. 2022).

To systematically assess the feasibility of these substitutions, three solvent sets were designed: Set 1 (chloroform + ethanol), Set 2 (2-MeTHF + methanol), and Set 3 (2-MeTHF + ethanol). This stepwise replacement allowed comparison of partial versus complete substitution relative to the standard Bligh & Dyer method. The modified procedures yielded slightly lower amounts of total lipid, decreasing from 11.14 ± 0.5% (conventional) to 10.97 ± 0.5% (Set 1), 10.26 ± 0.7% (Set 2), and 9.89 ± 0.5% (Set 3). This reduction is attributable to differences in solvent polarity and extraction capacity. Subsequently, lipid extracts were analysed by GC-FID, and the fatty acid profiles are summarized in Table 2. Results revealed clear differences across sets. Set 2 and Set 3 enhanced PUFA recovery (30.67% and 35.20%, respectively) compared to the conventional method (28.44%), indicating that 2-MeTHF improved solubility and partitioning of long-chain unsaturated fatty acids (Fig. 1b). Notably, set 3 provided the highest total PUFA content (35.20%), within which ω-3 fatty acids accounted for 19.80%, surpassing the conventional method while maintaining balanced levels of MUFA (44.32%) and SFA (20.47%).

**Table 2:** Lipid yield and fatty acid composition of conventional vs modified Bligh & Dyer methods. Values were expressed as g/100 g of total lipid extract

Parameters (%)	Conventional Bligh & Dyer method	Modified Bligh & Dyer method		
		Set- 1 Chloroform + Ethanol	Set–2 2-MeTHF + Ethanol	Set–3 2-MeTHF + Ethanol
Lipid Yield	11.14 ± 0.5	10.97 ± 0.5	10.26 ± 0.7	9.89 ± 0.5
Total SFA	36.53	33.90	24.75	20.47
Total MUFA	34.94	36.58	44.48	44.32
Total PUFA	28.44	29.42	30.67	35.20

### Concentration of PUFA by urea complexation

Building upon the green-solvent modified Bligh & Dyer extraction, the lipid fractions obtained were further concentrated via urea complexation. This method exploits the preferential crystallization of saturated and monounsaturated fatty acids in urea inclusion complexes, thereby enriching the filtrate in non-complexed polyunsaturated fatty acids(Hayes et al. 1998). To evaluate the effect of crystallization intensity, varying urea-to-free fatty acid (urea:FFA) molar ratios (2:1, 4:1, 6:1, and 8:1) were tested, and the resulting fractions were analysed by GC-FID (Table 2). A clear pattern emerged. At 2:1, PUFA content was 37.69%, increasing to 40.99% at 4:1 and peaking at 56.03% at the 6:1 ratio (Fig. 1c), representing nearly a twofold enrichment compared to the crude extract (Table 3). A further increase to 8:1 resulted in a decline to 41.35%, indicating that excessive urea may begin to co-precipitate unsaturated fatty acids, thereby reducing efficiency.

**Table 3:** Comparative GC-FID analysis of crude lipid extract and Fatty acid concentrations of various urea-to-free fatty acid (urea:FFA) molar ratios (2:1, 4:1, 6:1, and 8:1)

Fatty Acids	Conventional Bligh & Dyer method	Modified Bligh & Dyer method	2:1 (Urea: FFA)	4:1 (Urea: FFA)	6:1 (Urea: FFA)	8:1 (Urea: FFA)
SFA	36.53	20.47	22.60	13.94	24.13	20.09
MUFA	34.94	44.32	39.62	44.86	19.74	38.55
PUFA	28.43	35.20	37.69	40.99	56.03	41.35
ω-3	20.16	19.80	0.81	17.98	49.86	26.46
ω-6	8.27	15.40	8.06	23.01	6.21	14.89
Trans Fats	< 0.1	< 0.1	< 0.1	< 0.1	< 0.1	< 0.1

The ω-3 fraction showed a sharper enrichment: from only 0.81% at 2:1 to 17.98% at 4:1, then reaching 49.86% at 6:1, before declining to 26.46% at 8:1. The saturation fatty acid (SFA) fraction fell gradually (22.60% → 20.09%), while MUFA dropped steeply to 19.74% at 6:1, confirming their strong tendency to form urea complexes. The ω-6 fraction fluctuated moderately (6.21–23.01%), consistent with partial inclusion and exclusion based on structural flexibility. Together, these results indicate that lipid yield decreased modestly during green-solvent extraction, while urea complexation led to an increase in total PUFA from 28.44% to ~ 56.0% and ω-3 from 20.16% to ~ 49.86% (at 6:1) urea-to-FFA molar ratio.

### Biological activities of crude lipid and concentrated PUFA

Polyunsaturated fatty acids (PUFAs), particularly ω-3 PUFAs, have been widely recognized for their broad spectrum of biological activities, including antimicrobial (Paul et al. 2018), antioxidant (Yoshida et al. 2003), wound healing (McDaniel 2021), and anti-inflammatory effects (Calder 2017). In line with these reported benefits, we validated the biological relevance of the extracted fractions by subjecting both crude lipid and concentrated PUFA to antibacterial, antioxidant, cytotoxicity, and wound healing assays. To further establish their therapeutic potential, the anti-inflammatory assay was performed exclusively on the concentrated PUFA fraction, owing to its superior enrichment in ω-3 fatty acids.

### Antimicrobial activity (MIC analysis)

The antimicrobial activity of the crude lipid and concentrated PUFA fractions were evaluated against four representative bacterial strains as shown in the Table 4. Conventional agar well- or disc-diffusion assays were not employed because lipids and oil-based extracts exhibit poor diffusion in solid media, often leading to underestimation of antimicrobial activity (Hossain 2024). Instead, growth inhibition was determined using a broth microdilution approach with spectrophotometric quantification. Accordingly, antimicrobial activity is reported as MIC₅₀, defined as the lowest concentration producing ≥ 50% inhibition of bacterial growth.

**Table 4:** Minimum Inhibitory Concentration (MIC_50_) of crude lipid and PUFA-enriched fraction against selected MTCC strains

Bacterial species (MTCC strain)	Crude lipid (mg·mL⁻^1^)	PUFA-enriched (mg·mL⁻^1^)	Antibiotic control (mg·mL⁻^1^)
*Staphylococcus aureus*	128 ± 2.7	24.4 ± 0.3	AMP: 0.0005
*Pseudomonas aeruginosa*	64 ± 1.8	8.3 ± 0.1	CIP: 0.00025
*Escherichia coli*	No inhibition ≤ 256	No inhibition ≤ 256	CIP: 0.00025
*Vibrio alginolyticus*	No inhibition ≤ 256	No inhibition ≤ 256	CIP: 0.00025

Values are presented as Mean ± SD where n = 3; Antibiotic controls: AMP = Ampicillin, CIP = Ciprofloxacin

The PUFA-enriched fraction exhibited lower MIC₅₀ values than the crude lipid extract for the susceptible bacterial strains tested. Against *S. aureus*, the PUFA fraction inhibited growth at 24.4 ± 0.3 mg·mL⁻^1^ (MIC₅₀), whereas the crude lipid required 128 ± 6.5 mg·mL⁻^1^(MIC₅₀). Similarly, for *P. aeruginosa*, the MIC_50_ dropped from 64 ± 4.2 mg·mL⁻^1^ in crude lipid to 8.3 ± 0.1 mg·mL⁻^1^ in the PUFA fraction, representing an approximately eightfold improvement. These reductions suggest that selective concentration of PUFA enhanced antibacterial potency within complex lipid mixtures. By contrast, *E. coli* and *V. alginolyticus*, exhibited no inhibition at concentrations up to 256 mg·mL⁻^1^, consistent with literature reporting Gram-negative resistance due to outer membrane permeability barriers and efflux systems (Baker et al. 2018).

### Antioxidant activity

**Fig. 2:**
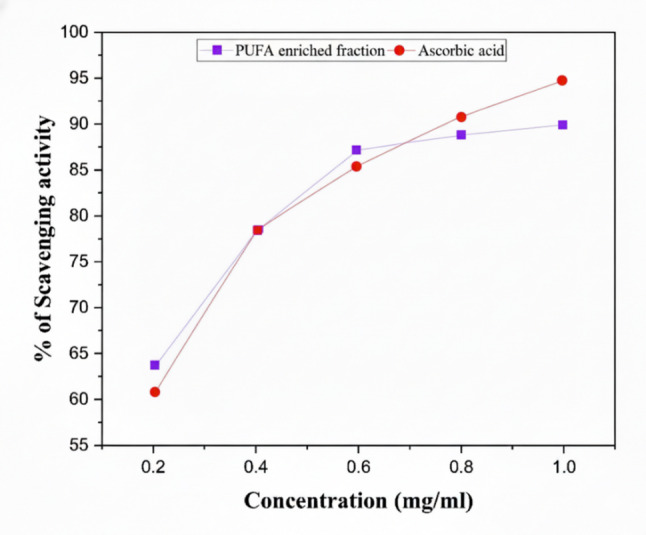
DPPH Free Radical Scavenging Activity of standard and PUFA-enriched fraction

The antioxidant activity of *Catla catla* concentrated PUFA fraction was assessed using the DPPH radical scavenging assay (Fig. 2). The results showed a clear, concentration-dependent increase in radical scavenging capacity, rising from 61.0 ± 1.0% at 0.2 mg mL⁻^1^ to 88.9 ± 0.9% at 1.0 mg mL⁻^1^. The activity increased nearly linearly between 0.2 and 0.6 mg mL⁻^1^, reaching ~ 87% at 0.6 mg mL⁻^1^, after which a gentle plateau was observed, suggesting saturation of available DPPH radical sites. Importantly, since > 60% inhibition was already achieved at 0.2 mg mL⁻^1^, the IC₅₀ can be estimated to be below 0.2 mg mL⁻^1^, indicating very high radical-scavenging efficiency.

### Cytotoxicity assessment

**Fig. 3:**
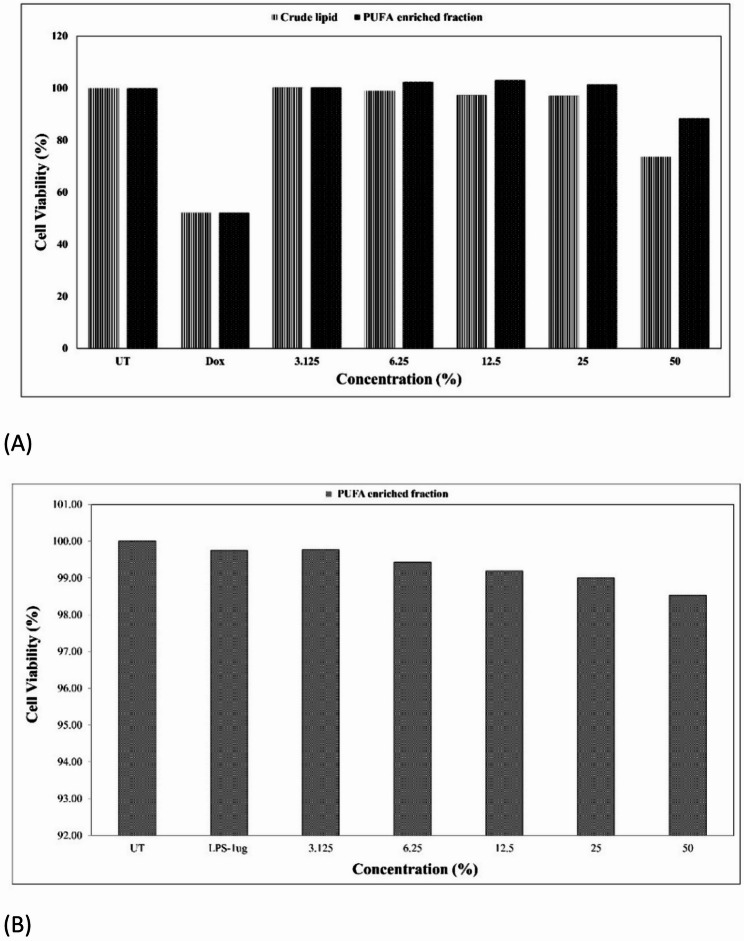
Cytotoxicity of *Catla catla* lipid fractions (**A**) Viability of NHEK-293 cells after 24 h exposure to crude and PUFA-enriched fractions, with untreated (UT) and doxorubicin (Dox; 1 µg/mL) as controls. (**B**) Viability of RAW 264.7 macrophages treated with PUFA-enriched fraction, with UT and lipopolysaccharide (LPS;1 µg/mL) as controls. Data are mean ± SD (n = 3)

The cytotoxic potential of *Catla catla* lipid fractions was first assessed using the MTT assay on NHEK-293 and RAW 264.7 cell lines to establish safe working concentrations (Fig. 3). Both crude lipid and PUFA-enriched fractions demonstrated a concentration-dependent effect on cell viability. In NHEK-293 cells, crude lipid fractions maintained ≥ 80% viability up to 25% concentration, whereas the PUFA-enriched fraction showed higher tolerance, with ≥ 85% viability even at 50% concentration (Fig. 3B). A one-way ANOVA followed by Tukey’s post hoc test indicated no significant difference (*p* > 0.05) between treated and untreated controls up to 25% concentration for the crude lipid fraction and up to 50% for the PUFA-enriched fraction, confirming their non-cytotoxic nature.

In RAW 264.7 macrophages, similar trends were observed, where viability remained above 80% across all tested concentrations of the PUFA-enriched fraction (Fig. 3B), while the crude fraction showed a mild but statistically significant decrease (*p* < 0.05) at the highest dose (50%). Importantly, no IC₅₀ value was reached within the tested range, indicating the absence of overt cytotoxicity.

### Wound-healing assay

**Fig. 4:**
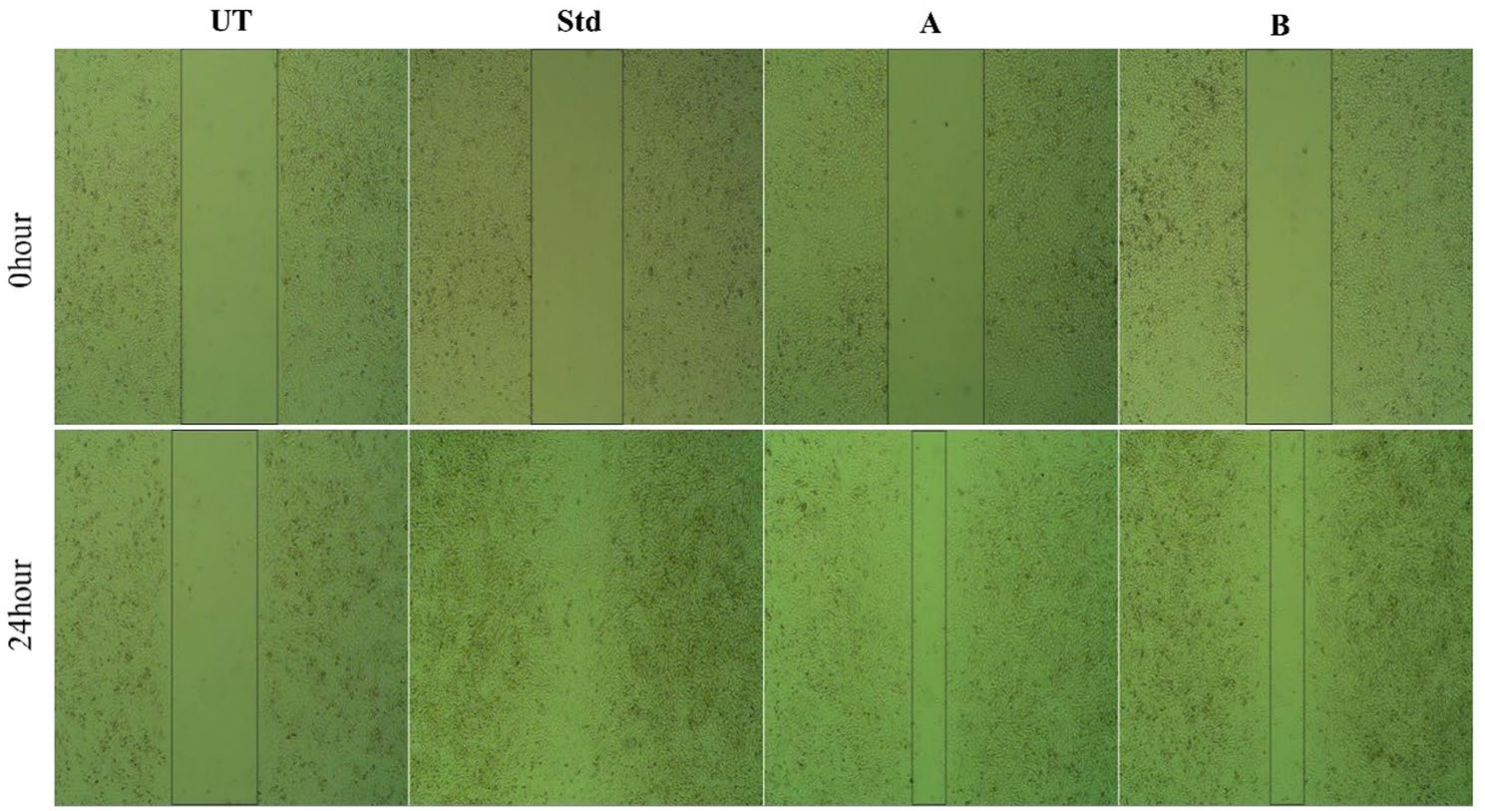
Wound-healing assay of *Catla catla* lipid fractions on NHEK-293 cells. Representative images at 0 h and 24 h showing scratch closure in untreated control (UT), standard (Std, Cipladine 50 µg/mL), (**A**) crude lipid, and (**B**) PUFA-enriched fraction

The wound-healing or scratch assay is a widely accepted in vitro model of tissue repair, as it mimics cell migration into a wound gap and provides a simple, reproducible measure of regenerative potential (Liang et al. 2007; Shekatkar et al. 2025).NHEK-293 epithelial cells were selected because they readily form confluent monolayers suitable for migration studies and have been used in recent scratch assays to evaluate bioactive compounds. Both crude lipid and PUFA-enriched fractions promoted wound closure within 24 h, with the PUFA fraction exhibiting slightly greater closure efficiency than crude lipid at equivalent concentrations (Fig. 4). Quantitative ImageJ analysis revealed that crude lipid achieved ~ 76.01% closure, whereas the PUFA-enriched fraction reached ~ 78.79% closure under identical conditions.

A one-way ANOVA followed by Tukey’s post hoc test indicated that both fractions produced significantly greater closure (*p* < 0.05) compared with the untreated control, while the difference between the crude and PUFA fractions was not statistically significant (*p* > 0.05)*.* Since cytotoxicity screening confirmed both fractions were non-toxic at the tested doses (≥ 80% viability at 25% for crude and up to 50% for PUFA), the enhanced wound healing can be attributed to improved migratory activity rather than differential survival. The slightly higher closure observed for the PUFA fraction strongly supports its regenerative potential and demonstrates the functional advantage of selective enrichment.

### Anti-inflammatory assay

The anti-inflammatory assay was carried out to evaluate whether PUFA-enriched fractions could modulate the inflammatory phase of wound healing. Inflammation is a critical early step in tissue repair, and when excessive or unresolved, it can delay closure and lead to chronic wounds (Wilkinson and Hardman 2020). In the scratch assay, both crude lipid and PUFA-enriched fractions promoted epithelial migration, but the enriched fraction achieved significantly greater closure efficiency. Based on this superior performance, only the PUFA-enriched fraction was advanced to anti-inflammatory testing, as effective wound healing requires not only cell migration but also resolution of inflammation.

To address this, RAW 264.7 macrophages were used instead of NHEK-293 epithelial cells. Unlike epithelial models, macrophages are central regulators of wound-associated inflammation, producing both pro-inflammatory cytokines (e.g., TNF-α, IL-1β) and anti-inflammatory mediators (e.g., TGF-β1, IL-10). RAW 264.7 is a well-established immune model for LPS-stimulated inflammation, widely used for nutraceutical and lipid bioactivity testing (Calder 2015). Using this cell line allowed us to specifically evaluate whether *Catla catla* PUFA fractions could rebalance inflammatory responses.

**Fig. 5:**
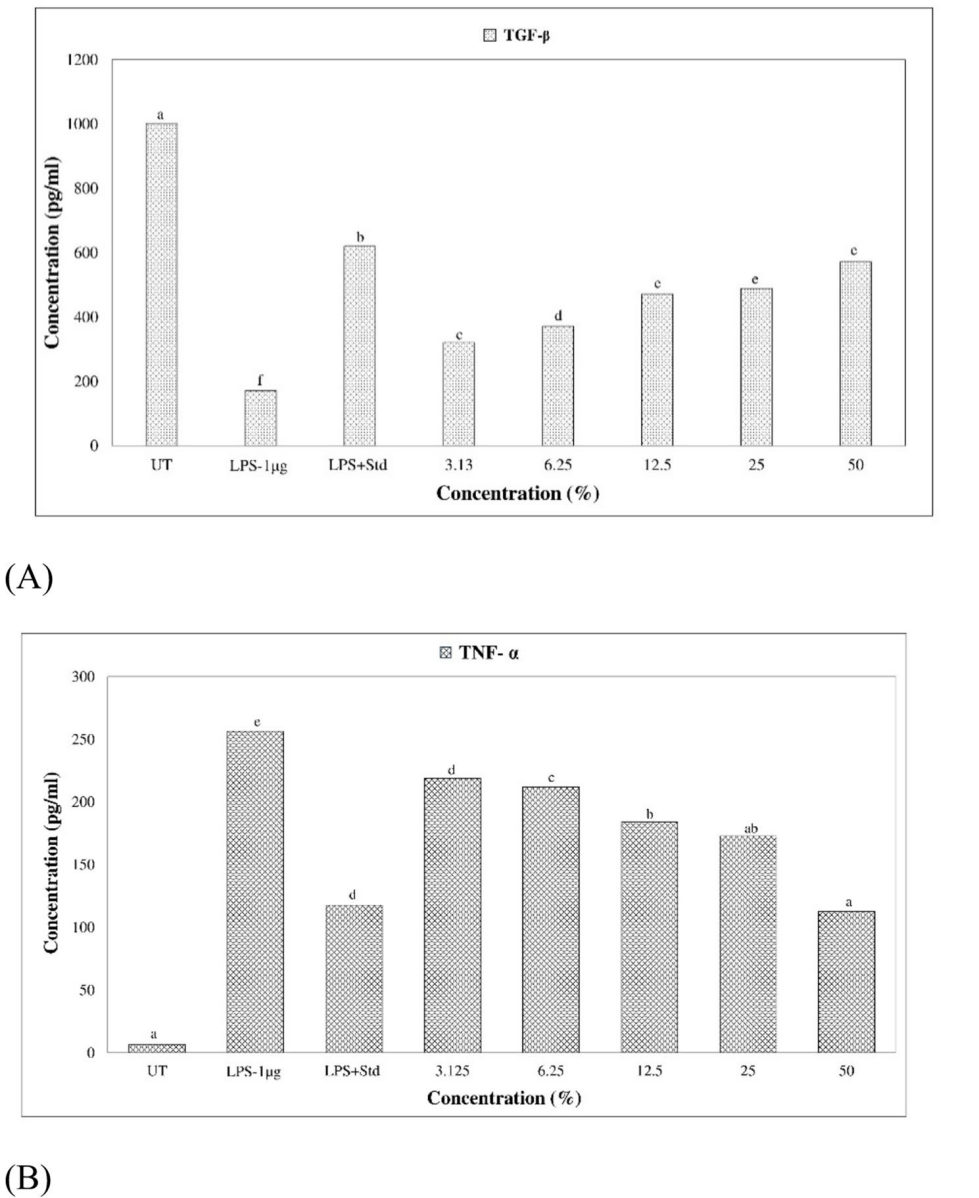
Effect of the PUFA-enriched *Catla catla* lipid fraction on inflammatory cytokine levels in LPS-stimulated RAW 264.7 macrophages: (**A**) TGF-β₁ and (**B**) TNF-α. Bars represent mean ± SD (n = 3). Different letters indicate significant differences (*p* < 0.05, one-way ANOVA with Tukey’s post hoc test). UT, untreated control; LPS, lipopolysaccharide (1 µg/mL); Std, standard drug (cordycepin 30 µg/mL)

In our experiments, LPS challenge increased TNF-α secretion to 256.45 pg/mL while reducing TGF-β1 to 171.89 pg/mL, confirming a strong inflammatory phenotype. Treatment with PUFA-enriched fractions produced a dose-dependent reversal. At 50% concentration, TNF-α levels decreased to 112.68 ± 1.61pg/mL, while TGF-β1 increased to 571.52 ± 11.98 pg/mL approaching the modulatory effect of the standard drug, cordycepin (Fig. 5A, B). Statistical evaluation through one-way ANOVA with Tukey’s post hoc test confirmed a significant reduction in TNF-α (*p* < 0.01) and a marked elevation in TGF-β₁ (*p* < 0.001) relative to the LPS-only control. This dual action—suppressing a pro-inflammatory marker while simultaneously enhancing an anti-inflammatory cytokine—demonstrates the fraction’s broad immunoregulatory activity.

## Discussion

### Extraction yield of lipids

The yield of 11.14 ± 0.5% observed using the Bligh & Dyer protocol in this study is notably higher than the 7.56% reported by Hasekar et al. (2020) from *Catla catla* viscera, and the 9.9% reported by Crexi et al. (2009) from assorted fish by-products. These findings establish this solvent-based approach as a benchmark for both neutral and polar lipid recovery. The relatively high yield in the present study is consistent with the use of viscera, which are inherently richer in lipid-storing tissues compared to muscle or head fractions.

For UAE, Similar trends have been documented by Mai et al. (2023) who observed 43.5% lipid recovery from trout muscle, and Dave et al. (2024) reported 10.55% from *Clarias magur* viscera, representing a 9.17% increase compared to wet rendering. Nevertheless, in viscera-rich tissues, the dense protein and connective tissue matrix can entrap lipids and promote emulsification, which may limit the overall efficiency of UAE despite its strong disruptive potential (Zhou et al. 2021).

The autoclave-assisted extraction yielded the highest recovery in the present investigation (16 ± 0.7%), contrasting with the much lower value (5.37 ± 0.22%) reported by Lakmini et al. (2022) from *Thunnus albacares* heads by following. This disparity highlights the importance of tissue type in lipid extraction studies. Viscera, unlike heads or muscle, contain lipid-rich organs such as liver and intestines that inherently accumulate fats. This distinction is supported by Cao et al. (2024), who reported that viscera consistently provide higher lipid yields than other body parts across both freshwater and marine fishes.

Beyond extraction method, species type and ecological conditions also influence the percentage of yields. *Catla catla*, a widely cultured tropical freshwater carp, is generally classified as a medium-fat fish based on muscle lipid content, yet its viscera contain substantially higher lipid levels, resembling fatty tissue within the species (Swapna et al. 2010)**.** Comparatively, lean fish such as cod, tilapia yield < 2% lipids in muscle, whereas fatty fish species like sardine and salmon yield up to 20% (Nava et al. 2023). Cold-water fish typically accumulate higher levels of PUFAs to maintain membrane fluidity at low temperatures, whereas tropical species like *Catla catla* exhibit lipid profiles influenced strongly by feed and culture practices (Voronin et al. 2021). This trend extends to other Indian major carps like *Labeo rohita* viscera contained nearly ten times more lipids than muscle and around 1.5 times more than head tissue (Swapna et al. 2010). Such observations reinforce that viscera represent a robust and underutilized source of lipids across related species. Apart from this, wild fish often show seasonal variability, but cultured fishes raised under controlled conditions provide more consistent lipid yields and fatty acid profiles, increasing the reliability of these findings and their future applicability (Yusoff et al. 2024). These factors make viscera of cultured *Catla catla* a promising substrate for valorization. While pooling of viscera prior to extraction reduced inter-individual variability and enabled direct comparison of extraction methods, this approach limits assessment of biological variation between individual fish. However, yield alone cannot estimate nutritional quality. Because, the primary aim of this work is to obtain PUFA-enriched fractions, so the choice of the most suitable extraction method can be concluded in the following Sect. (3.2) through analysing fatty acid composition.

### Characterization of fatty acids

UAE and autoclave-assisted extractions are discussed here primarily as comparative benchmarks to contextualize lipid yield and fatty acid distribution, rather than as optimized strategies. The Bligh & Dyer method provided the most balanced profile, with a total PUFA content of 28.44%, of which ω-3 fatty acids accounted for 20.16%, and substantial enrichment of EPA (9.48%) and DHA (6.82%).This distribution reflects the ability of the chloroform–methanol system to recover both neutral and polar lipid fractions efficiently (Alfio et al. 2021). Collectively, UAE favoured MUFA and ω-6 fatty acids (20.24 g/100 g, mostly linoleic acid, C18:2n6), autoclaving increased SFA at the expense of PUFA, while Bligh & Dyer achieved the highest recovery of ω-3 PUFA.

The PUFA values obtained (6.05–20.16%) are broadly consistent with earlier reports on *Catla catla* ranging from 19.8 to 21.6% (Andhale et al. 2017; Mahindarathna et al. 2024) and in muscle tissue, 14.3–18.7% depending on water (Paul et al. 2018). These variations likely reflect (i) the use of visceral tissue, which accumulates long-chain PUFA for reproductive functions (Zhong et al. 2007), and (ii) method-dependent differences in fatty acid distribution. Because oxidative stability indicators such as peroxide value or TBARS were not measured, differences in ω-3 PUFA profiles among extraction methods are interpreted in terms of compositional outcomes rather than oxidative degradation or preservation mechanisms. Although Bligh & Dyer gave the highest ω-3 yields, its reliance on chloroform and methanol poses safety and environmental concerns. Therefore, the next stage of this study explored a green-solvent-modified B&D protocol to ensure sustainable PUFA recovery.

### Green-solvent modified Bligh & Dyer extraction

Although, the B&D method is widely regarded for its efficiency in solvent-based lipid recovery, its reliance on chloroform and methanol presents environmental and health hazards. In this study, these solvents are replaced with eco-friendly solvents (2-MeTHF and ethanol). Despite a modest reduction in total lipid yield, these solvent systems were associated with an increased proportion of nutritionally relevant ω-3 PUFA suggesting a compositional shift toward PUFA-rich fractions. Previous studies reported ethanol- or 2-MeTHF/ethanol-based lipid extraction from microalgae and oilseeds (Bourgou et al. 2021; Yi et al. 2023); however, their application to fish viscera remains limited in the literature. Recent work on black soldier fly larvae has shown that 2-MeTHF can enhance recovery of phospholipids and free fatty acids compared with non-polar solvents (Smets et al. 2021). In aquatic viscera, ω-3 PUFA are often enriched in polar lipid fractions rather than exclusively in neutral storage lipids. In the present study, the reduced total lipid yield relative to the classical Bligh & Dyer method may reflect preferential extraction of polar lipid fractions, while excluding a proportion of neutral lipids. These observations indicate that replacing toxic solvents with green alternatives is feasible for fish viscera without loss of PUFA quality. Such findings broaden the scope of sustainable extraction practices and support future applications of green chemistry in aquatic lipid valorization.

### Concentration of PUFA by urea complexation

The pronounced increase in PUFA andω-3 fatty acids content observed at a 6:1 urea:FFA molar ratio indicates compositional enrichment under these conditions. These outcomes are consistent with classical observations by Ratnayake et al. (1988), who reported maximal enrichment at ~ 3:1 ratios, and with subsequent reports by Crexi et al. (2012) and Lin et al. (2014), which demonstrated comparable optimal ranges (3:1–7:1) depending on source oil and crystallization temperature**.** Recently, Vázquez et al. (2022) further reported enhanced PUFA concentration via urea adduct methods to remove SFAs more effectively. In fish oil systems, Eskandari et al. (2024)demonstrated urea complexation as a useful route to separate PUFA from kilka oil.

As the urea-to-free fatty acid ratio increased, the yield decreased progressively from 74 (at 1:1) down to 32% (at 6:1). This reduction in mass was accompanied by an increase in PUFA proportion. The ‘peak’ phenomenon of ω-3 fraction exhibited a sharp enrichment trajectory, peaking at 49.86% at the 6:1 ratio. This suggests a plateau behavior at higher urea levels, a limitation observed in similar studies (Liu et al. 2006). Excessive urea ratios led to the re-solubilization of saturated fatty acids due to the increased volume of crystallization solvent required, as reported by Setyawardhani et al. (2018). This resulted in the retention of saturated fatty acids in the filtrate at the 6:1 ratio.

Urea complexation is widely applied as a relatively simple fractionation approach compared to more complex separation techniques (Rollin et al. 2023). Importantly, the sequential strategy, wherein green-solvent extraction followed by urea complexation enabled PUFAenrichmentfrom an underutilized fish waste resource while minimizing the use of toxic solvents. This integrated approach aligns with the broader objective of valorizing aquatic by-products for nutraceutical applications. Mass recovery across the urea complexation steps was not quantified in the present study and should be addressed in future work aimed at process optimization and scale-up.

### Antimicrobial activity (MIC analysis)

The enhanced antibacterial performance of PUFA-rich fractions is consistent with earlier findings that eicosapentaenoic acid (EPA) and docosahexaenoic acid (DHA) exert activity against Gram-positive bacteria, including *S. aureus* (Le and Desbois 2017). Mechanistically, these long-chain polyunsaturated fatty acids disrupt bacterial membranes, altering phospholipid composition and integrity, which compromises cell viability (Herndon et al. 2020). The MIC₅₀ values observed in the present study indicate moderate but selective antibacterial activity of PUFA-enriched fractions, particularly against Gram-positive bacteria. The lack of effect against *E. coli* and *Vibrio* further reflects the structural resilience of Gram-negative bacteria, which restrict PUFA penetration through their outer membrane.

These findings highlight two key points: (i) PUFA enrichment significantly enhances antibacterial activity relative to crude lipids, especially against Gram-positive bacteria, and (ii) the moderate, concentration-dependent inhibition supports the potentialof PUFA-rich fractions as nutraceutical bioactives and functional food components rather than antibiotic substitutes.

### Antioxidant activity

The concentrated PUFA fraction exhibited potent free radical scavenging activity, achieving nearly 89% inhibition at 1.0 mg mL⁻^1^. This finding compares favourably with literature reports. For example, Akmal and Roy (2017) observed an IC₅₀ of 0.148 mg mL⁻^1^ for fish liver oil, but required concentrations up to 4 mg mL⁻^1^ to achieve ~ 96% inhibition. In contrast, concentrated PUFA fraction in the present study achieved nearly 89% inhibition at just 1.0 mg mL⁻^1^, highlighting its strong antioxidant potential at relatively low concentrations. Similar plateau kinetics have been widely reported in natural antioxidants, where scavenging activity rises steeply at lower concentrations and subsequently stabilizes as radical binding sites become saturated (Mendonça et al. 2022; Suhag et al. 2025)**.**

The comparison with the standard control further validates the activity profile. While the standard exhibited slightly stronger scavenging at the highest tested concentration (~ 95% at 1.0 mg mL⁻^1^), concentrated PUFA fraction performed equally or better in the intermediate concentration range (0.2–0.6 mg mL⁻^1^), suggesting that the lipid matrix and enriched PUFA content contributed significantly to free radical neutralization. This is consistent with previous reports that long-chain polyunsaturated fatty acids, particularly EPA and DHA, act as electron donors and chain-breaking antioxidants, thereby reducing oxidative stress (Nasopoulou and Zabetakis 2012). The strong DPPH scavenging activity observed here demonstrates that *Catla catla* fish oil, even prior to further functional validation, represents a promising nutraceutical candidate. Its antioxidant potential complements its antibacterial effects and underpins its broader application in functional foods and bioactive formulations aimed at combating oxidative damage.

### Cytotoxicity assessment

The findings confirm the non-cytotoxic nature of both the crude and PUFA-enriched lipid fractions at working concentrations ≤ 50% (v/v), with the PUFA fraction demonstrating enhanced biocompatibility. The improved tolerance of the PUFA-enriched fraction compared to crude lipid likely reflects removal of minor contaminants and oxidized residues during enrichment, yielding a cleaner, more biocompatible composition. Comparable observations have been reported for marine lipid concentrates rich in ω-3 PUFAs, which exhibit low cytotoxicity in non-tumorigenic lines such as NHEK-293 and macrophage models while retaining bioactivity (Freiría-Martínez et al. 2025; Jang et al. 2022). These results provided the rationale for selecting ≤ 50% dilution for subsequent wound-healing and anti-inflammatory assays, ensuring that functional outcomes reflected biological modulation rather than cell death.

### Wound-healing assay

The observed enhancement in wound closure by both lipid fractions underscores their regenerative potential, which can be attributed to fatty acid mediated promotion of epithelial cell migration. This observation is consistent with the established role of ω-3 PUFAs in accelerating epithelial repair, whereby they enhance membrane fluidity, promote keratinocyte migration, and support generation of pro-resolving lipid mediators that drive wound resolution (Serhan and Levy 2018). Comparable effects have been observed in prior studies, where PUFA-rich marine oils enhanced keratinocyte migration and angiogenesis in vitro and in vivo (Ontoria-Oviedo et al. 2022), and where pro-resolving lipid mediators such as lipoxins directed fibroblast proliferation and scratch-wound closure (Herrera et al. 2015). The superior effect of the PUFA fraction over crude lipid in our study therefore demonstrates that selective enrichment not only improves antimicrobial and antioxidant properties but also augments functional bioactivity directly relevant to wound healing.

### Anti-inflammatory assay

The PUFA-enriched fraction from *Catla catla* demonstrated marked anti-inflammatory activity in RAW 264.7 macrophages, effectively shifting the cytokine balance from pro-inflammatory (TNF-α dominant) toward anti-inflammatory (TGF-β₁ dominant) profiles. This modulation mirrors physiological pathways observed during the resolution phase of wound healing and underscores the importance of PUFAs in restoring immune homeostasis. When compared with earlier published reports, these values are highly encouraging. For instance, PUFA-enriched fraction supplementation in RAW 264.7 cells has been shown to lower LPS-induced TNF-α by ~ 40–50% and increase anti-inflammatory cytokines in a similar magnitude (Monmai et al. 2020; Jang et al. 2022)**.** Our PUFA fraction reduced TNF-α by ~ 44% and more than doubled TGF-β1 compared to LPS-only, aligning closely with or even exceeding reported effects of other marine lipid extracts. Such potency suggests that PUFA enrichment from *Catla catla* viscera generates fractions with comparable or superior bioactivity to well-studied marine oil systems. In the present study, the inflammatory model was specifically designed to evaluate modulation of LPS-induced cytokine responses; basal cytokine release in macrophages treated with lipid fractions in the absence of LPS stimulation was therefore not quantified and should be addressed in future investigations to further delineate intrinsic immunomodulatory effects.

Overall, by extending validation from epithelial scratch assays to macrophage inflammatory models, we establish that PUFA-enriched fractions not only accelerate closure but also promote resolution of inflammation, addressing two key phases of wound repair. These findings underscore the therapeutic potential of *Catla catla*-derived PUFAs as multifunctional agents for wound-healing applications. Although individual ω-3 fatty acids such as EPA and DHA were quantified chemically, all the bioactivity assays conducted in the present study were performed using the PUFA-enriched fraction as a whole. Therefore, the observed biological effects reflect the combined action of enriched fatty acids rather than isolated contributions of individual fatty acids.

### Circular economy context and societal relevance

*Catla catla* viscera are currently undervalued by-products within freshwater aquaculture systems typically disposed of or sold at minimal prices. The valorization framework explored in this study suggests that recovery of crude and PUFA-enriched lipids from viscera could enhance resource utilization and support more sustainable use of aquaculture by-products. These considerations indicate potential socio-economic relevance, particularly for small-scale processing units. Recovered lipids may find applications ranging from bulk uses (such as animal feed, soaps, and biodiesel) to higher-value nutraceutical, pharmaceutical, and cosmetic formulations, depending on the degree of refinement. Quantitative assumptions, indicative cost components, and value estimations are provided in the supplementary Table (S1).

Through institute–industry linkages, local cooperatives or processing units could potentially be engaged to supply crude lipids to bulk industries or collaborate with nutraceutical and pharmaceutical companies for PUFA-enriched fractions. Such pathways illustrate how sustainable valorization may extend beyond academic demonstration to support waste reduction and rural livelihood opportunities, these economic considerations are preliminary and based on indicative assumptions; detailed techno-economic analysis and pilot-scale validation are required before drawing conclusions on commercial feasibility within a circular bioeconomy framework.

The present study demonstrated the sustainable valorization of *Catla catla* viscera, an underutilized aquaculture by-product, through green solvent extraction coupled with urea complexation to yield PUFA-enriched fractions with significant antimicrobial, antioxidant, wound-healing, and anti-inflammatory activities. This dual focus on environmental sustainability and functional validation highlights the potential of viscera-derived lipids as valuable nutraceutical and biomedical resources. Importantly, the economic assessment revealed that simple valorization protocols can transform low-value waste into high-value products, providing new livelihood opportunities and aligning with circular bioeconomy principles. Future research should extend this work to in vivo studies for confirming therapeutic efficacy, detailed toxicological evaluation, and exploration of formulation-based applications such as topical ointments, supplements, and functional foods. Additionally, pilot-scale demonstrations and institute–industry collaborations will be crucial to translate laboratory findings into scalable technologies, ensuring that the benefits of waste-to-wealth strategies are realized across aquaculture communities and broader society.

## Supplementary Information

Below is the link to the electronic supplementary material.

Supplementary Material 1

## Data Availability

All related data and methods are present in this paper. Additional inquiries can be addressed to the corresponding author.

## Abbreviations

PUFA: Polyunsaturated fatty acids
SFA: Saturated fatty acids
MUFA: Monounsaturated fatty acids
EPA: Eicosapentaenoic acid
DHA: Docosahexaenoic acid
2-MeTHF: 2-Methyltetrahydrofuran
GC-FID: Gas chromatography-flame ionization detection
UAE: Ultrasound-assisted extraction
AAE: Autoclave-assisted extraction
B&D: Bligh and Dyer
LPS: Lipopolysaccharide
TNF-α: Tumor necrosis factor-alpha
TGF-β1: Transforming growth factor-beta 1
MTT: 3-(4,5-Dimethylthiazol-2-yl)-2,5-diphenyltetrazolium bromide
DPPH: 2,2-Diphenyl-1-picrylhydrazyl
ELISA: Enzyme-linked immunosorbent assay
PBS: Phosphate-buffered saline
DMSO: Dimethyl sulfoxide
PBST: Phosphate-buffer saline with tween-20
FFA: Free fatty acids
FBS: Fetal bovine serum
NHEK-293: Normal human epidermal keratinocytes
DMEM: Dulbecco’s modified eagle medium
MTCC: Microbial type culture collection
MIC: Minimum inhibitory concentration

## Declarations

### AI and AI-assisted technologies

During the preparation of this work the authors used ChatGPT in order to refine the language and for grammatical error correction. After using this tool/service, the authors reviewed and edited the content as needed and take full responsibility for the content of the publication.
