# Supplementary material for: Sustainable valorization of fish viscera into omega-3 rich lipids and their functional validation
Source: AMB Express. 2026 Feb 20;16:35. doi: 10.1186/s13568-026-02029-1 (PMC13031517; doi:10.1186/s13568-026-02029-1)
Supplement: Supplementary file 1 — Supplementary Material 1 [file 13568_2026_2029_MOESM1_ESM.docx]

***
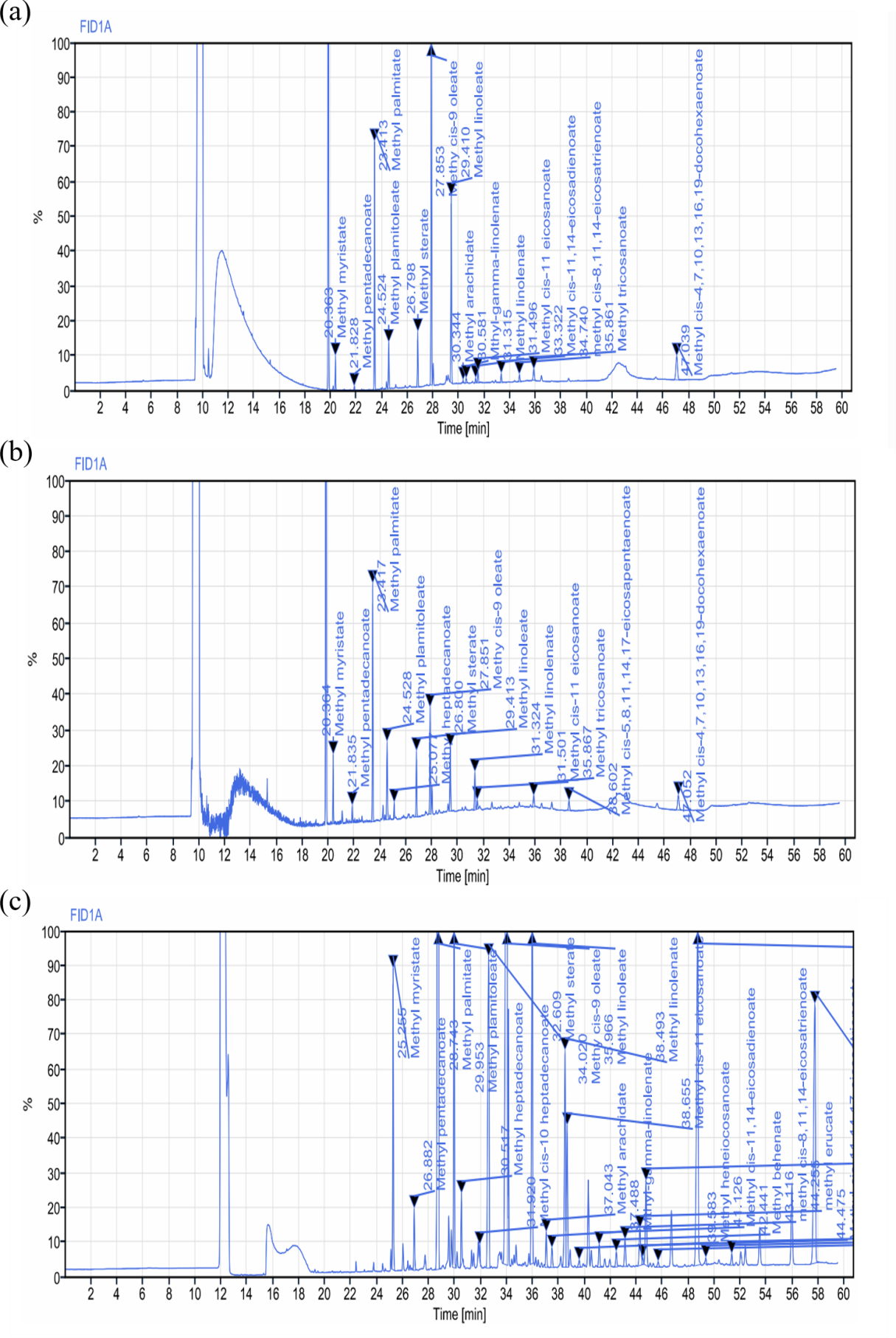
***

**Fig.S1 GC-FID chromatograms of lipid extracts obtained using ultrasound-assisted and autoclave-assisted extraction methods, included for comparative assessment of fatty acid distribution.**

**Table S1. Preliminary economic assumptions and indicative value estimation for PUFA valorization from Catla catla viscera**

| Item | Low estimate | High estimate |
| --- | --- | --- |
| Viscera recovered (kg per 1000 kg fish) | 100 | 180 |
| PUFA-enriched oil yield (kg) | 5 | 14 |
| Current value (raw viscera, ₹2/kg) | 200 | 360 |
| Potential value (PUFA oil, ₹3,000–5,000/kg) | 20,000 | 57,600 |
| Estimated processing cost (₹) | 23,000 | 30,500 |
| Net outcome (₹) | –10,000  (Net loss) | +27,100 |

Assumptions: average *Catla* weight 2.5–3.0 kg; viscera fraction 10–18%; lipid yield 10–16% with ~50% PUFA enrichment; nutraceutical PUFA oil price ₹3,000–5,000 per kg; solvent cost after 80% recovery ~₹140/L; manpower ₹600/day; energy and overhead ~₹10,000 per tonne batch.
